# Forecasting Influenza Epidemics in Hong Kong

**DOI:** 10.1371/journal.pcbi.1004383

**Published:** 2015-07-30

**Authors:** Wan Yang, Benjamin J. Cowling, Eric H. Y. Lau, Jeffrey Shaman

**Affiliations:** 1 Department of Environmental Health Sciences, Mailman School of Public Health, Columbia University, New York, New York, United States of America; 2 WHO Collaborating Centre for Epidemiology and Biostatistics, School of Public Health, Li Ka Shing Faculty of Medicine, The University of Hong Kong, Hong Kong Special Administrative Region, China; Duke University, UNITED STATES

## Abstract

Recent advances in mathematical modeling and inference methodologies have enabled development of systems capable of forecasting seasonal influenza epidemics in temperate regions in real-time. However, in subtropical and tropical regions, influenza epidemics can occur throughout the year, making routine forecast of influenza more challenging. Here we develop and report forecast systems that are able to predict irregular non-seasonal influenza epidemics, using either the ensemble adjustment Kalman filter or a modified particle filter in conjunction with a susceptible-infected-recovered (SIR) model. We applied these model-filter systems to retrospectively forecast influenza epidemics in Hong Kong from January 1998 to December 2013, including the 2009 pandemic. The forecast systems were able to forecast both the peak timing and peak magnitude for 44 epidemics in 16 years caused by individual influenza strains (i.e., seasonal influenza A(H1N1), pandemic A(H1N1), A(H3N2), and B), as well as 19 aggregate epidemics caused by one or more of these influenza strains. Average forecast accuracies were 37% (for both peak timing and magnitude) at 1-3 week leads, and 51% (peak timing) and 50% (peak magnitude) at 0 lead. Forecast accuracy increased as the spread of a given forecast ensemble decreased; the forecast accuracy for peak timing (peak magnitude) increased up to 43% (45%) for H1N1, 93% (89%) for H3N2, and 53% (68%) for influenza B at 1-3 week leads. These findings suggest that accurate forecasts can be made at least 3 weeks in advance for subtropical and tropical regions.

## Author Summary

Influenza causes high levels of morbidity, mortality, and economic burden. Accurate forecasts of epidemic timing and magnitude would provide public health sectors valuable advance information in support of the planning and deployment of intervention measures. Such forecast systems have been developed for temperate regions with seasonal winter epidemics (e.g., U.S. cities). In subtropical and tropical regions, however, influenza epidemics can occur throughout the year with varying epidemic intensity; this irregularity makes the generation of accurate forecasts more challenging. For this study we develop alternative forecast systems that are more adept at handling erratic non-seasonal epidemics, using state-of-the-art Bayesian inference methods in conjunction with an epidemiological model. Here we present these forecast systems and apply them to Hong Kong. During 1998–2013, Hong Kong saw 44 influenza epidemics caused by either the A(H1N1), A(H3N2), or B strain, and 19 aggregate epidemics caused by one or more of these influenza strains. The forecast systems are able to forecast both the peak timing and peak magnitude of these epidemics, including the 2009 pandemic. The results suggest that routine forecast of influenza epidemics in other subtropical and tropical regions is possible, as well as forecast of other infectious diseases sharing similar irregular transmission dynamics.

## Introduction

Influenza causes a significant public health burden worldwide. Recent studies have shown that reliable forecasts of influenza epidemics can be generated in real time [1–3]. Particularly, operational forecasts of influenza epidemics have been developed for temperate regions such as the continental U.S. [4,5] These efforts could be valuable in aiding planning and deployment of intervention measures (e.g., health promotion activities and the distribution of vaccines and antivirals). However, before operational forecasts can be routinely generated and expanded to other regions of the globe, these forecast systems need to be tested and optimized against epidemics observed in a variety of locales with diverse transmission dynamics.

In temperate regions, influenza transmits primarily during winter. This regularity allows the forecast systems to be initiated and optimized prior to the influenza season. For instance, our real-time forecast system for U.S. cities [1,4] is initialized at Week 40 each year, the first week that the U.S. Centers for Disease Control and Prevention (CDC) begin influenza activity surveillance. The forecast system is then continuously “trained” throughout the following weeks and months as additional observations are received and assimilated to inform the influenza transmission dynamics in that season. Model state variables, e.g., number of susceptible people and number of infected people, can be inferred through this recursive training process. These model state variables and parameters form the initial conditions of a forecast, which are critical for generating an accurate prediction.

**Fig 1:**
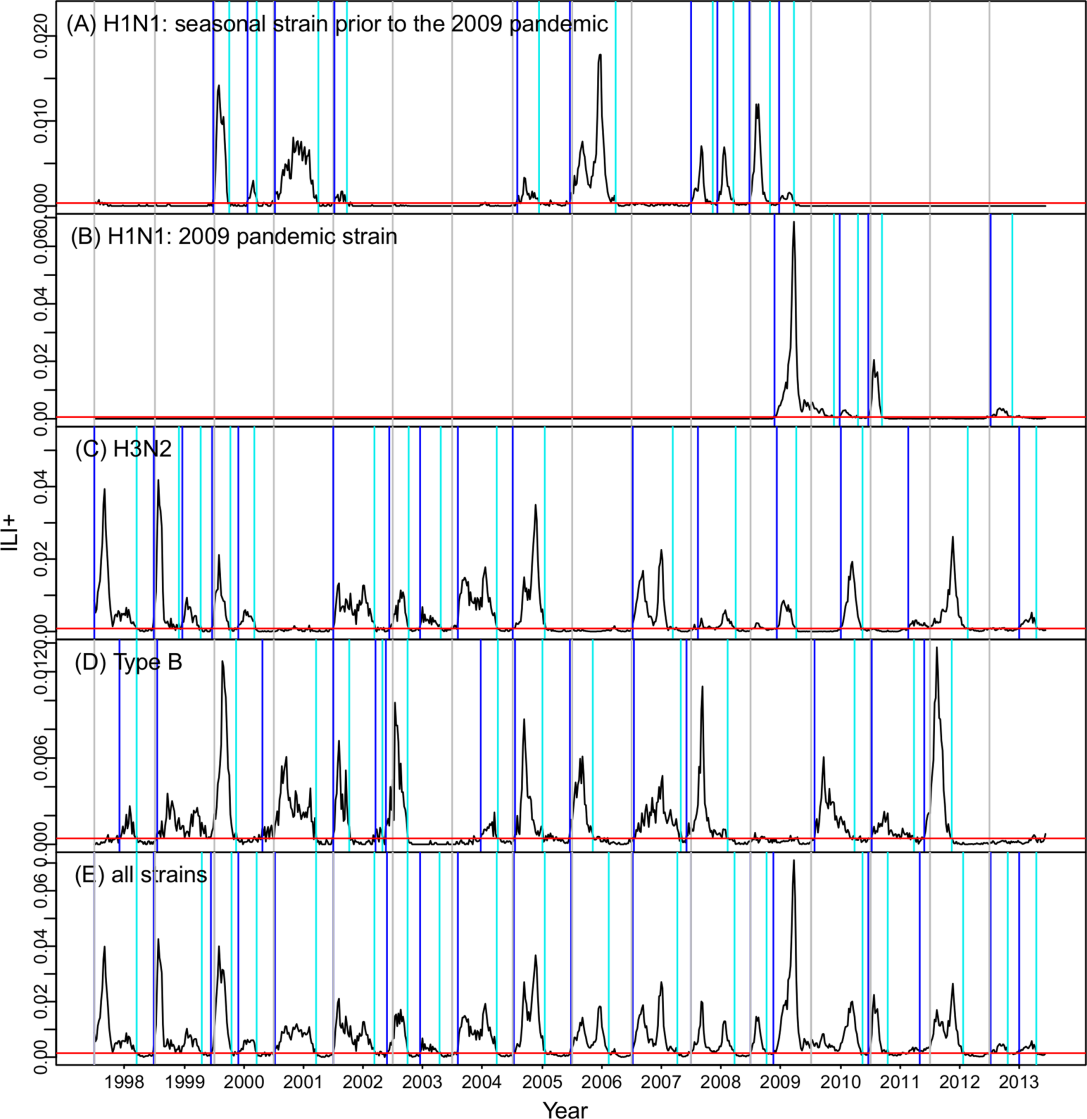
Time series of ILI+ for each strain: (A) seasonal A(H1N1), (B) pandemic A(H1N1), (C) A(H3N2), (D) Influenza B, and (E) all strains. Black lines are ILI+ observations; red horizontal lines are baselines; blue vertical lines are the identified onsets; cyan vertical lines are identified endings; grey vertical lines are year divisions.

In subtropical and tropical regions, however, the seasonal characteristics of influenza are more diverse. Hong Kong is one such area that experiences highly irregular influenza epidemics from year to year [6–8]. Hong Kong is located on the south coast of China, with a humid subtropical climate. It is one of the most densely populated cities, with a population of over seven million people and a population density of 6544 per km^2^ [9] (cp. 33.7 per km^2^ in the U.S. [10]). In addition, Hong Kong is highly connected with mainland China and other regions around the world, attracting over 50 million visitors per year [11]. This large influx of visitors may increase the importation of influenza cases and further facilitate local transmission. Due to these unique climatic and socioeconomic features, influenza epidemics in Hong Kong can persist year-round in one year, whereas one or more distinct epidemics can occur in another year (Fig 1). In addition, outbreak intensity, duration, and time from onset to the peak is more variable in Hong Kong than in temperate regions (S1 Fig). This irregularity poses challenges for operational influenza prediction. For instance, initialization of the system at the beginning of the season, as done for temperate regions, would not be possible. As such, it is not clear whether the same forecast system, proven to be valuable for temperate regions with regular epidemics, could be applied to generate forecasts in real time for subtropical and tropical regions.

Indeed, our initial attempt to forecast the Hong Kong influenza epidemics using the same system for U.S. cities proved unfruitful (S2 Fig). To overcome these challenges, we developed alternate forecast systems that are more adept at handling the seasonally erratic influenza transmission dynamics of the subtropics and tropics. Here we present these forecast systems and apply them to Hong Kong. The results are promising for forecasting influenza outbreaks in other subtropical and tropical regions, as well as other infectious diseases sharing similar irregular transmission dynamics.

## Materials and Methods

### Data

Weekly records of rates of influenza like illness (ILI) consultations in the community from the week ending 04 January 1998 to the week ending 15 December 2013, were reported by a sentinel surveillance network of approximately 50 outpatient clinics [7,8,12]. The Public Health Laboratory Services Branch in the Centre for Health Protection conducts laboratory testing of specimens provided by the ILI network and local hospitals for surveillance and diagnostic purposes. We obtained data on the weekly number of influenza-positive specimens by type and subtype, and the weekly number of specimens tested [7,8,12]. From these data streams we calculated the weekly ILI+ rate, a metric more precisely representing influenza infections [1,13–15]; specifically, ILI+ was calculated as the ILI rate multiplied by the viral detection rate for each strain individually or all strains combined (S1 Dataset). Forecasts were done separately for these 4 ILI+ time series, i.e., H1N1 (combining seasonal and pandemic H1N1), H3N2, influenza B, and the time series combining all influenza strains.

### Model-filter forecast systems

#### (1) Epidemic model

Unlike temperate regions where influenza epidemics recur regularly each winter, Hong Kong experiences epidemics throughout the year. The intrinsic factors contributing to this year-round transmission pattern, e.g., different transmission routes or cross-immunity between different A subtypes and/or Type B, as well as the extrinsic effects of humidity and other environmental conditions on influenza transmission in this subtropical region, are not well understood [16–18]. Consequently, for the modeling pursued here, we chose a simple susceptible-infected-recovered (SIR) model without humidity forcing rather than the humidity-forced SIRS model used in previous studies [1,13,19,20].

The SIR model simulates the numbers of susceptible and infected persons using the following equations:

$$\frac{dS(t)}{dt}=-\frac{R_{0}}{D}\cdot \frac{I(t)S(t)}{N}-\alpha$$ (1)

$$\frac{dI(t)}{dt}=\frac{R_{0}}{D}\cdot \frac{I(t)S(t)}{N}-\frac{I(t)}{D}+\alpha$$ (2)

where *S* is the number of susceptible persons, *t* is time in days, *N* is the population size, *I* is the number of infectious people, *α* is the rate of travel-related influenza case import into the model domain, *D* is the mean infectious period, and *R_0_* is the basic reproductive number. The term $\frac{R_{0}}{D}$ on the right hand side of Eqs 1 and 2 represents the influenza transmission rate. All model variables (i.e., *S* and *I*) and model parameters (i.e., *R_0_* and *D*) are estimated by the filters as described below.

#### (2) Mapping the SIR model to observations

Model simulations of infected persons and influenza incidence are discordant with the observations, ILI+. That is, the model represents influenza incidence on a per capita basis, or incidence rate, and includes asymptomatic and mildly symptomatic infections; ILI+ is an estimate of the number of symptomatic influenza infections per 100,000 patient visits. To address this discordance, we use a scaling factor, γ, to map the SIR model-simulated incidence rate to the ILI+ observation [1,21]. Briefly, the ILI+ observation estimates the probability that a person seeking medical attention, *m*, has influenza, i.e., *p*(*i*|*m*). By Bayes’ rule, the probability of a person contracting influenza during a given week, *p*(*i*) is

$$p(i)=\frac{p(m)}{p(m|i)}\cdot p(i|m)\approx \frac{p(m)}{p(m|i)}\cdot ({ILI}_{+})$$ (3)

The weekly incidence rate, ξ, estimated using the SIR model, is also an estimate of the probability of a person contracting influenza, *p*(*i*). Therefore, we can convert the model-simulated incidence rate to model-simulated ILI+, $\hat{{ILI}_{+}}$, per Eq 3 as follows:

$$\hat{{ILI}_{+}}=\frac{\xi }{\gamma }\approx \frac{p(i)}{\frac{p(m)}{p(m|i)}}$$ (4)

where $\gamma =\frac{p(m)}{p(m|i)}$ is the scaling factor. This scaling factor [1,21] is estimated by the filter in this study.

#### (3) Filters

Using the SIR model, we built two forecast systems to predict the Hong Kong ILI+ time series. The first system used the ensemble adjustment Kalman filter (EAKF) [22] for optimization; the second system used a particle filter with resampling and regularization (PF) [23]. Both filters adopt an ensemble approach. That is, an ensemble of model replicas (termed ensemble members for the EAKF or particles for the PF) is generated at initialization, and recursively updated at each prediction-update, i.e. filtering, cycle. The prediction step propagates the system forward to the next time step (e.g., from the current week to the next) generating a prediction using the SIR model; when a new observation arrives, the system (including all model variables and parameters) is updated per the filter algorithm (for more details see [1,13,19]). This updated ensemble provides a probability distribution for each model variable or parameter. The mean variable/parameter estimate can be computed based on the ensemble; the EAKF computes the mean as a weighted average between the prediction and the observation, while the PF computes the mean using the updated particle weights, which are derived from a likelihood function.

Both model-filter forecast methods include a ‘training’ process and a forecast step. The ‘training’ process comprises iterative prediction-update cycles as described above; it allows the filter to assimilate all observations up to the week of forecast and recursively optimize the model to obtain more authentic initial conditions prior to the forecast. The forecast step integrates the SIR model forward tens of weeks using the variables and parameters estimated from the training process.

Due to the irregular timing of influenza epidemics in Hong Kong, both forecast systems were run continuously from the first record (i.e., Week 1 of 1998) to the last record (i.e., Week 50 of 2013). (Previously, for seasonal influenza in temperate regions, we have reinitialized model training at the beginning of each season, e.g., Week 40 of the year in the U.S.). This long Hong Kong time series created challenges for both filters used here; during the later half of the time series, the PF suffered from particle impoverishment while the EAKF suffered from filter divergence [24]. To rejuvenate the SIR-PF system, we applied space re-probing (SR), a technique developed to prevent particle impoverishment during the filtering process [20]. Basically, the SR method randomly replaces the model variables/parameters (in this study, *S*, *R_0_*, *D*, and *γ*) within a small fraction of trajectories, i.e. particles, at each filtering cycle; in so doing, it expands the state space covered by the filter through the introduction of outlying trajectories. To prevent the EAKF from filter divergence, we reinitialized the SIR-EAKF system once filter divergence was detected (i.e., when the posterior diverged from an observation by over 20%); in addition, we applied adaptive covariance inflation to the EAKF [25,26]. Using these techniques, the PF was run with 3,000 particles and the EAKF was run with 500 ensemble members. Each week, following assimilation of the latest observation, a forecast was generated for the following 40 weeks (i.e., weekly 40-week forecasts). The first forecast was made after assimilating records from the first 3 weeks (i.e., Weeks 1–3 of 1998).

The priors for the state variables and parameters in the model-filter frameworks were drawn from uniform distributions: *S*~U[20%*N*, 80%*N*], for the initial population susceptibility with *N* = 1×10^5^ as the population size in the SIR model (note that *N* is scalable to the actual population size of interest), *R_0_*~U[0.6, 2.2], for the basic reproductive number, and *D*~U[0.5, 7] days, for the infectious period. The systems were seeded with 0–50 infected persons (i.e., *I*). In our past studies [1,13], the scaling factor γ was fixed based on trials from a range of values and values ranging from 2 to 15 had good predictive ability [1]. In this study, we included the scaling factor as a parameter of the forecast system and allowed it to be inferred by the filter; we used a broad prior distribution of γ for both forecast systems, i.e., γ~U [1, 10]. This choice of prior distribution reflects *a priori* belief that the probability a person seeks medical attention when infected with influenza (including asymptomatic and mild, symptomatic infections) is generally lower than that of a person seeking medical attention for any reason (e.g., cardiovascular diseases), which is consistent with previous studies suggesting low consultation rates for influenza [21,27]. Note, the model simulated states and parameters are free to go outside the prior range should the filters, particularly the EAKF, migrate to an expanded state space through filtering of the observations.

### Testing and evaluation of the two forecast systems

Each forecast system setting was used to simulate and forecast the H1N1, H3N2, B, or all strains combined ILI+ time series; each start-to-end forecast run was repeated 100 times to account for random effects from system initialization. The forecast systems using the two filters were then evaluated based on (1) accuracy predicting the phase or gross activity (i.e., epidemic or dormant period) and (2) accuracy predicting specific metrics, namely the onset, peak timing, peak magnitude, and duration of individual epidemic.

We defined the onset as the first of three consecutive weeks with ILI+ records exceeding a prescribed baseline. The ILI+ baselines were chosen as the 40% quantile of the non-zero ILI+ records for each influenza strain, or the first quartile of the non-zero ILI+ records for all influenza strains combined. Inclusion of only non-zero ILI+ records in this calculation focuses the definition of onset on periods when a strain is circulating; the 40% quantile or 25% quantile of the remaining non-zero records define epidemic periods. Results using alternate baselines, e.g. the 33% quantile, produced similar results; however, some epidemics were not well delineated using this lower threshold (e.g., two adjacent epidemics could be classified as one epidemic). We defined the ending of an epidemic as the first of two consecutive weeks with ILI+ below the baseline following an onset. The period between an onset and its respective ending was defined as an epidemic; however, only those events with an ILI+ record three times or more above baseline were considered, i.e. intermittent small spikes were excluded. Time periods other than epidemics were defined as dormant periods.

The first aforementioned evaluation (i.e. predicting the gross phase) was intended to test whether the forecast system can accurately predict upcoming epidemic events while not predicting spurious epidemics during a dormant phase. The forecast was assessed against the entire duration of an event (i.e., a dormant period or epidemic period as defined above). A phase prediction was deemed accurate if the predicted epidemic trajectory included an epidemic during the predicted period; similarly, it was deemed accurate if there was no predicted epidemic during a dormant period. For example, for a forecast made at Week 10, within a dormant period lasting from Week 5 to Week 30, a phase prediction is deemed accurate if the forecast time series over Weeks 5–30 does not include two consecutive weeks exceeding the baseline, but inaccurate if otherwise.

The second evaluation was used to assess whether a forecast system can accurately predict the timing of onset, the peak timing (i.e., the week with the maximum ILI+), the peak magnitude (i.e., the maximum ILI+), and the epidemic duration. All these specific metrics are of potential interest to public health officials for planning influenza-related intervention measures. The accuracy of these metric forecasts was evaluated by comparing the target and the predicted metric. If the week of forecast initiation was within a dormant period and an epidemic was predicted during the following 40 weeks, the predicted epidemic would be evaluated against the metrics of the next observed epidemic (i.e., timing of onset, peak, ending, and peak magnitude); if the week of forecast initiation was within an epidemic period, and a second epidemic was predicted during the 40-week forecast, the predicted epidemic would be evaluated against the current epidemic episode.

Two evaluation standards were adopted. For the stricter standard, predictions of the week of onset, peak timing, or duration were deemed accurate if they exactly matched observations, and predicted peak magnitude was deemed accurate if it fell within ±20% of the observed ILI+ value. For the looser standard, predictions of epidemic onset or peak within ±1 week of observation, duration within ±2 weeks of observation, and peak magnitude within ±50% of the observation were deemed accurate.

### Simple analog forecast

To test whether the filter methods outperform a naïve method, we also performed a simple analog forecast predicting the peak timing for the four ILI+ time series. The times from the onset to the peak for each epidemic over the entire study period were compiled from each time series; these historical records formed a database of time-to-peak for each strain or all strains combined (S1 Fig). For each time series, a weekly forecast was generated when ILI+ of the week exceeded the baseline (same as defined above), by randomly drawing a time-to-peak record from the corresponding database. A prediction was deemed accurate if the predicted peak was within ±1 week of observation. One hundred random forecasts were sampled for each week of each time series. Forecast accuracy was tallied over all samples and compared to the filter methods.

## Results

### Identifying epidemic episodes for each strain

With the prescribed baselines and definitions of onset and ending, we identified 14 epidemics of H1N1 (including 10 epidemics of seasonal H1N1 during 1998–2009 and 4 epidemics of the pandemic H1N1 since 2009), 16 epidemics of H3N2 and 14 epidemics of influenza B during 1998–2013. For the combined strain time series, for which multiple concurrent epidemics of co-circulating strains could overlap and be counted as one single epidemic, there were 19 influenza epidemics during 1998–2013. Fig 1 shows these time series along with the onset and ending of each epidemic.

### Modeling of the ILI+ time series

The combined system of the SIR model and either of the two filter methods comprises a state-space, or hidden Markov, model that allows estimation of unobserved, or latent, state variables (e.g., population susceptibility *S*) [28]. Both filters adjust the SIR model variables (e.g., numbers of susceptible and infected people) and parameters (e.g., the basic reproductive number *R_0_* and infectious period *D*) using observations through a recursive filtering process. For instance, at the beginning of an epidemic, a filter may adjust the susceptibility upwards in light of an increase in incidence. By doing so, it is able to accommodate the dynamics of the system, e.g., increased population susceptibility when a new strain begins to circulate, despite the fact that the SIR model does not include susceptible replenishment. In effect, the filters partially compensate for model misspecification.

Both model-filter methods were able to faithfully recreate each of the ILI+ time series (S3 Fig). In addition, estimates of the model variables and parameters (S4, S5 and S6 Figs), as recursively updated at each filtering cycle over the course of a simulation, were used to initiate the weekly forecasts of future influenza incidence.

### Forecast accuracy for gross epidemic activity

The forecast system predictions of gross activity were first evaluated for sensitivity and specificity. This assessment tests whether a particular forecast system could accurately predict upcoming epidemics in time while not predicting spurious epidemics during a dormant phase.

**Fig 2:**
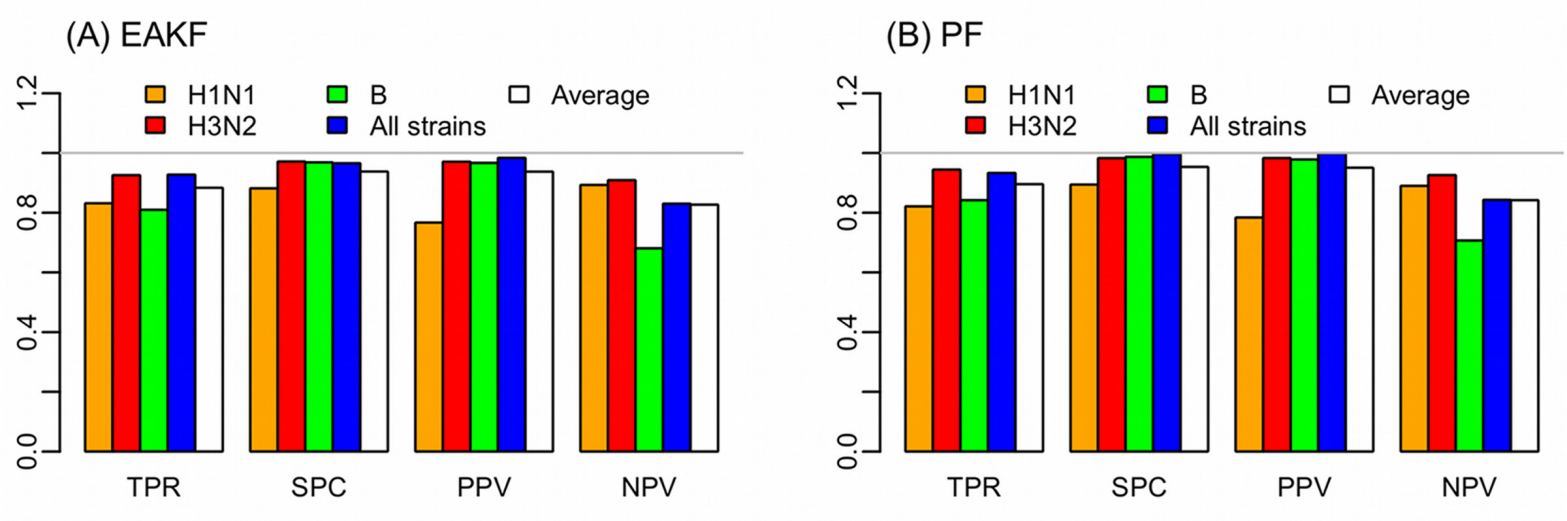
Accuracy predicting gross epidemic activity. Four measures, sensitivity (i.e., true positive rate, TPR), specificity (i.e., true negative rate, SPC), precision (i.e., positive predictive value, PPV), and negative predictive value (NPV) are shown for (A) the SIR-EAKF and (B) the SIR-PF forecast system. Results are tallied over forecast of H1N1 (orange), H3N2 (red), Type B (green), all strains combined time series (blue), and all forecasts (white).

Fig 2 shows the sensitivity (i.e., true positive rate), specificity (i.e., true negativity rate), precision (i.e., positive predictive value), and negative predictive value for the two forecast systems. Both forecast systems can accurately detect/predict an ongoing epidemic (sensitivity>~80%) and do not falsely predict epidemics during dormant periods (specificity >~90%). Tallied over all weekly forecasts, the PF had slightly higher sensitivity (90% vs. 88%) and specificity (95% vs. 94%) than the EAKF. For both filters, the sensitivity and specificity vary by strain; forecasts for H1N1 and Type B in general had lower sensitivity and specificity (e.g., for the EAKF, sensitivity of 83% for H1N1 and 81% for influenza B vs. 93% for H3N2). Supplemental S1–S4 Movies present the forecasts for each of the three strains and all strains combined epidemics at each week.

### Forecast accuracy for epidemic onset, peak timing, peak magnitude, and duration

**Fig 3:**
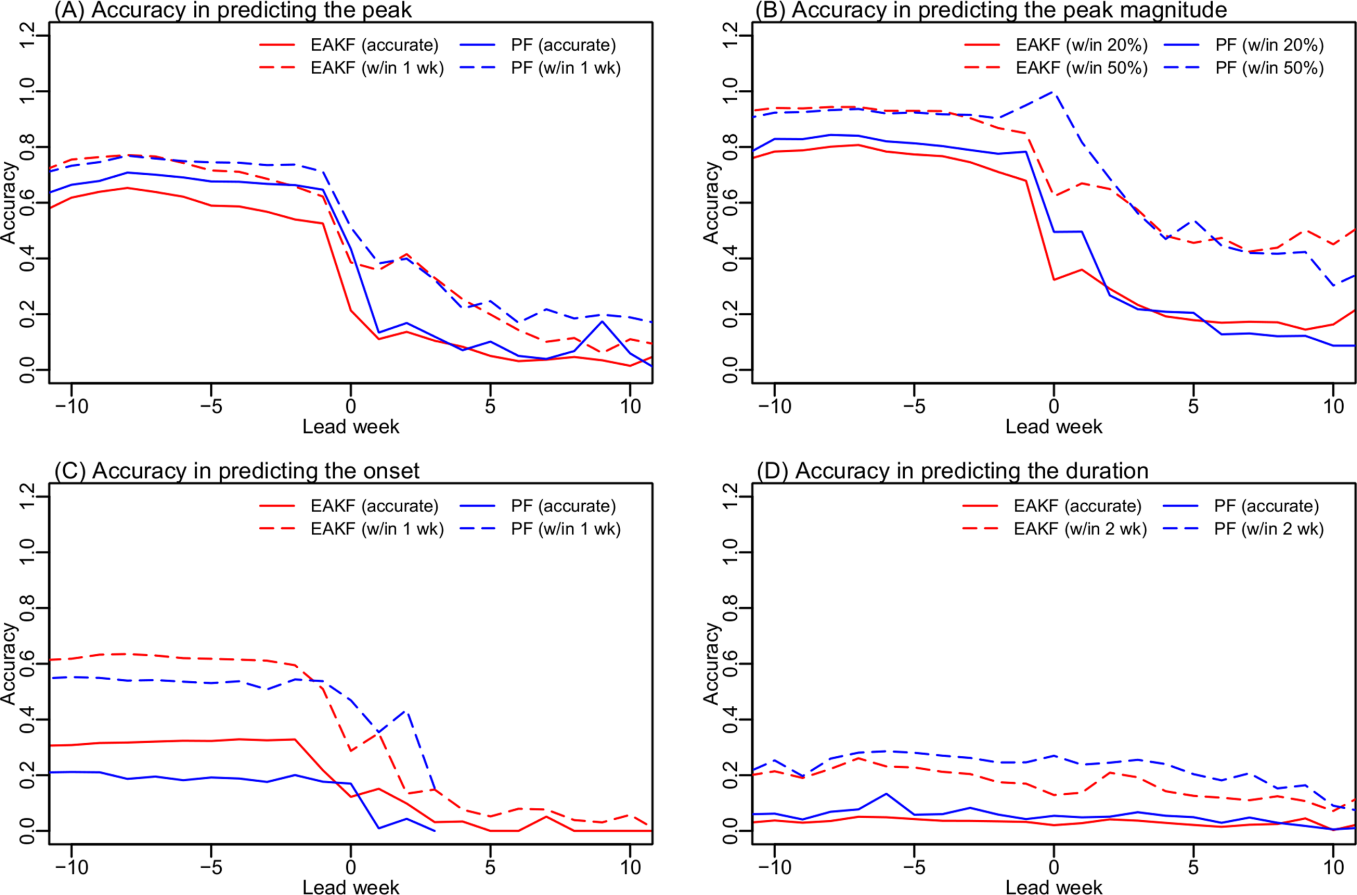
Accuracy predicting outbreak peak timing (A), peak magnitude (B), onset (C), and duration (D). Accuracy was calculated over all forecasts (332,400 for each setting of the forecast system). This analysis includes the forecasts for seasonal H1N1, the 2009 pandemic H1N1, H3N2, B and all strains combined. Results are shown for the EAKF (red) and the PF (blue), evaluated using two standards (solid vs. dashed lines, as specified in the parentheses). On the x-axis, positive leads indicate that a peak is forecast in the future; negative leads indicate that a peak is forecast in the past; a 0 week lead indicates that a peak is forecast as the same week of forecast. Leads are relative to the predicted peak for forecasts of the peak timing, peak magnitude, and duration, and relative to the predicted onset for forecasts of onset timing.

Fig 3 presents prediction accuracy for epidemic onset, duration, peak timing, and peak ILI+ magnitude for both forecast systems. These are tallied for all forecasts—individual strain and all strains—a total of 332,400 weekly forecasts (i.e., 831 weekly forecasts for each strain × 4 strains × 100 runs). Here we focus our analysis on *predicted* lead weeks ranging from -10 to 10 weeks; a positive lead (e.g. 2 wk) indicates the event (e.g., the epidemic peak or onset) is predicted to occur 2 weeks in the future from the time of forecast initiation; a 0 wk lead indicates the event is predicted to occur at the time of forecast initiation; and a negative lead, say -3 wk, indicates the event is predicted to have occurred 3 weeks prior to the forecast initiation. Forecasts with negative lead times may appear counterintuitive; however, accurate prediction that an event has passed is an important capability of a forecast system. In regions experiencing year-round influenza transmission, such as Hong Kong, multimodal epidemics, i.e. epidemics with multiple crests, are common (Fig 1). A forecast initiated after a smaller crest but preceding the overall peak may mistakenly identify that smaller crest as the peak and predict that the peak has passed, i.e. an inaccurate forecast with negative lead. Conversely, an accurate forecast with negative lead indicates that no spurious future increase in incidence is predicted. Therefore, forecast accuracy at negative leads also represents the ability of the forecast system to predict future epidemic trajectories.

**Fig 4:**
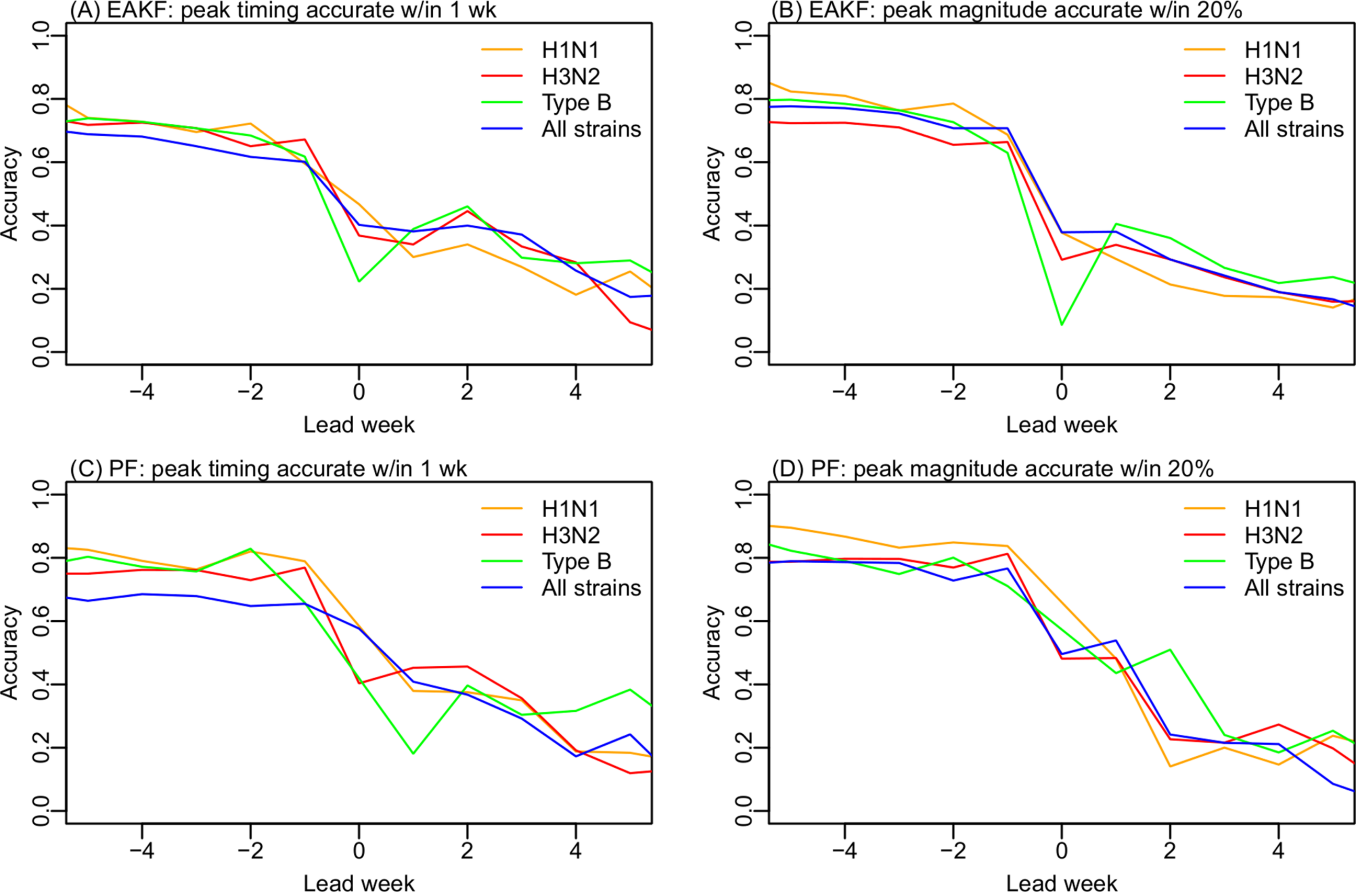
Performance of the SIR-EAKF (A and B) and the SIR-PF (C and D) for individual strains predictions of peak timing (A and C) and magnitude (B and D).

Forecast accuracy differs by filter, timing of forecast initiation, and the metric as well as the time series being forecast. Tallied over all forecasts, the PF in general produces more accurate predictions of peak timing (within ±1 wk of observation), peak magnitude (within ±20% of observation), and epidemic duration (within ±2 wk of observation), while the EAKF is more accurate predicting onset timing (within ±1 wk of observation, Fig 3). However, neither filter was able to predict outbreak onset or duration in advance of these events. Given the great irregularity in epidemic timing in Hong Kong, this outcome is not surprising. Some epidemics last for over a year in Hong Kong (Fig 1 and S1 Fig); in such instances, even 10 weeks after the outbreak peak, the conclusion of the epidemic remains difficult to predict accurately. Both filters were able to more accurately predict peak timing and peak magnitude by individual strain than for the aggregate time series combining all circulating strains (Fig 4). This finding suggests that strain specific observations may provide cleaner signals that enable more accurate forecast using the single strain SIR model.

Overall, the SIR-PF predictions of peak timing and peak magnitude outperformed those of the SIR-EAKF forecast system (Fig 3). The performance of the SIR-PF forecast system is promising. Summarized over all weekly forecasts: (1) for the peak timing predictions (within ±1 wk of observation), accuracy was 37% for peaks 1–3 wk in the future, 51% for a current peak (0 wk lead), and increased to 73% for peaks 1–2 wk in the past (1–2 wk lag); (2) for the peak magnitude predictions (within ±20% of the observed peak ILI+), accuracy was 37% for peaks 1–3 wk in the future, 50% for a current peak, and increased to 78% for peaks 1–2 wk in the past (1–2 wk lag).

**Fig 5:**
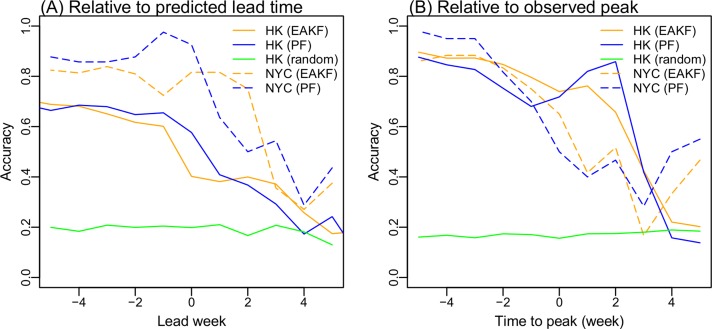
Comparison of forecast accuracy for Hong Kong (HK) and New York City (NYC) using the EAKF and PF filters, as well as random sampling from historical records for HK. Forecast accuracy was evaluated by grouping predictions based on (A) predicted lead time (i.e. how far in the future the peak is predicted) or (B) actual forecast week relative to the observed peak. Positive leads indicate that a peak is forecast in the future; negative leads indicate that a peak is forecast in the past; a 0 week lead indicates that a peak is forecast for the week of forecast initiation.

We also compared with the ILI+ forecasts generated for New York City, a temperate city with population size and density comparable to Hong Kong (8.4 million vs 7.2 million; 10,725 people/km^2^ vs 6,544 people/km^2^), to those of Hong Kong. Over the 2003–2013 period and using epidemic curves aggregated for all circulating strains [20], peak prediction accuracy for Hong Kong is lower (Fig 5A). This is likely due to the more complex influenza transmission dynamics in Hong Kong, e.g., longer outbreak duration and multiple peaks in a year (S1 Fig). Indeed, this gap disappeared when forecast accuracy was evaluated by timing relative to the observed peak, as opposed to the predicted lead week (Fig 5B). For those forecasts initiated 3 weeks prior to the observed local peak or thereafter, accuracies for Hong Kong were comparable to or higher than those for New York City (Fig 5B). Moreover, when compared with a simple analog method, both filter methods clearly were more accurate (Fig 5).

### Forecast certainty

Both filters used here adopt an ensemble approach (see Materials and Methods). The ensemble, i.e., the collection of model replicas, provides an estimated distribution for each model variable and parameter, as well as forecast epidemic trajectories. Previous forecast studies for the U.S. indicate that forecast accuracy increases when the variation within the forecast ensemble decreases [1,13,19]. This relationship can be used to calibrate forecast certainty and thus segregate more and less accurate forecasts in real time. That is, an expected accuracy for a real-time forecast, similar to the chance of precipitation in a weather forecast, can be derived based on this relationship. In real-time operation, the accuracy of a forecast cannot be verified until the epidemic has concluded; therefore, the expected accuracy, if reliable, provides forecast users, such as public health officials, much richer information.

**Fig 6:**
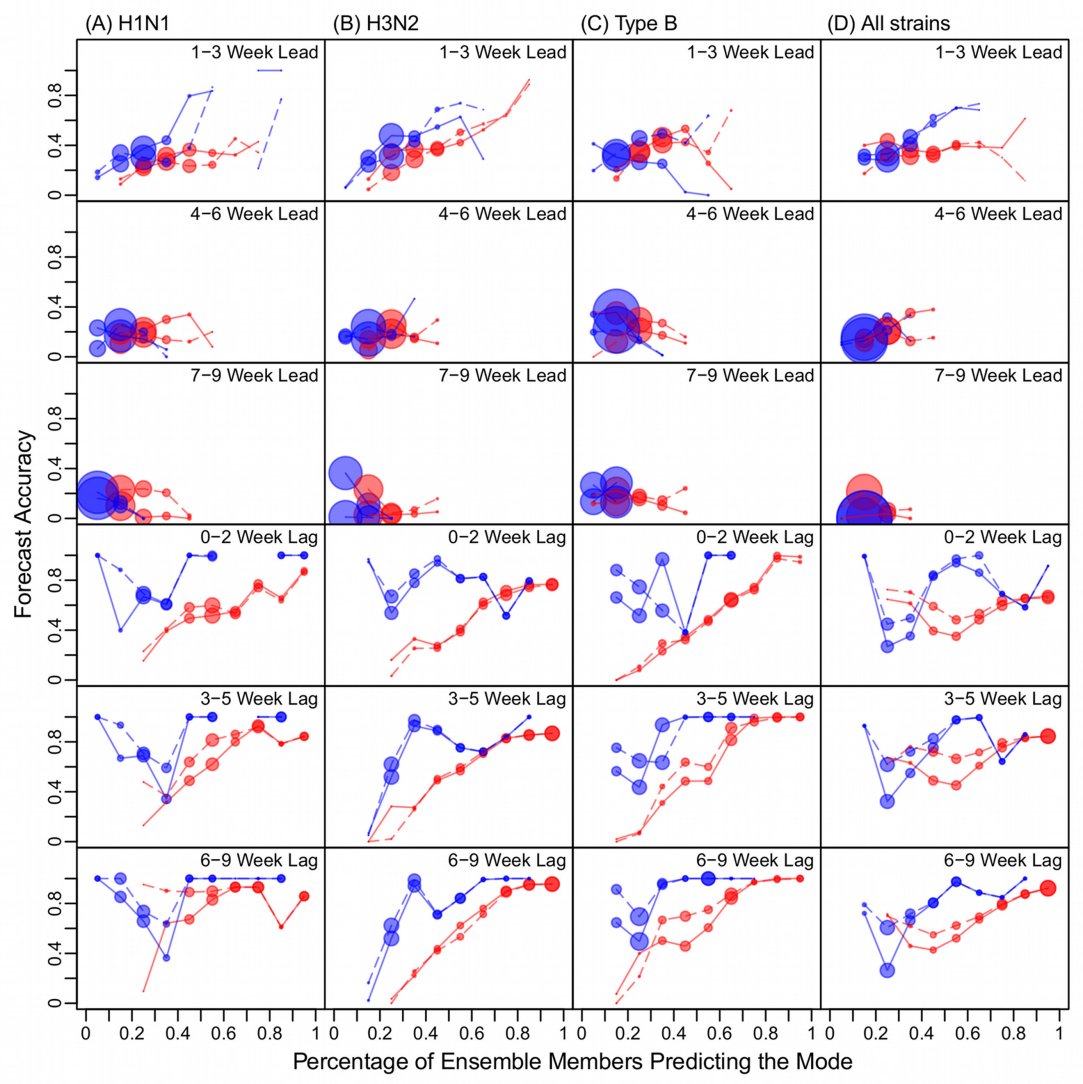
Forecast accuracy versus ensemble spread. The ensemble spread is presented as the percentage of ensemble members predicting the mode peak week (PEMPM). Red lines are generated using the EAKF and blue lines using the PF; solid lines show forecast accuracies for peak timing (within ±1 week of observation); dashed lines show forecast accuracies for peak magnitude (within ±20% of observation). The filled circle size associated with each PEMPM bin represents the fraction of forecasts within each lead category.

Here we determined whether such a relationship holds for the Hong Kong forecasts. As in our previous study [13], we found that the ensemble spread can be represented by the percentage of ensemble members predicting the mode (PEMPM). As defined previously [13], PEMPM is the percentage of the most frequently predicted outcome (i.e. the mode) among all predicted outcomes. The PEMPM increases when the agreement among ensemble members increases; it thus provides a measure of the variation within a forecast ensemble. Fig 6 shows this relationship for forecasts of peak timing using the SIR-EAKF and SIR-PF. Forecast accuracy does tend to increase as the PEMPM increases (i.e., the ensemble members are more in agreement), particularly for forecasts with a predicted peak 1–3 weeks in the future (i.e., 1–3 wk lead) at the forecast initialization or in the past (i.e., 0–2 wk lag, 3–5 wk lag, or 6–9 wk lag). This relationship is more robust for H3N2, H1N1, or all strains combined than for influenza B for forecasts with positive leads (Fig 6A, 6B and 6D vs. 6C, 1^st^ and 2^nd^ rows, solid lines). For the more virulent and dominant H3N2 strain, the forecast accuracy for peak timing at 1–3 wk lead increased steadily up to 93% as the PEMPM increased to 80–90% (Fig 6B, 1^st^ row).

In addition, a similar relationship appears between the accuracy of the predicted peak magnitude and the PEMPM of predicted *peak timing* (Fig 6, dashed lines). Further, this relationship for peak magnitude forecast accuracy was also clear for influenza B (Fig 6C, dashed lines). For H3N2, the forecast accuracy for peak magnitude at 1–3 wk lead increased up to 89% as the PEMPM increased to 80–90% (Fig 6B, 1^st^ row). These relationships indicate that the forecast systems are able to accurately predict both peak timing and peak magnitude at least 3 weeks in advance.

## Discussion

In previous work, we developed forecast systems for influenza, which have demonstrated predictive skill when applied to U.S. cities [1,13,19]. These studies suggest operational forecasts can be achieved and have motivated the generation of real-time influenza forecasts [4]. However, unlike the regular seasonal epidemics in temperate regions such as the U.S., epidemics in subtropical and tropical regions are highly irregular [29–32]. The Hong Kong influenza incidence time series, for instance, create challenges not seen in seasonal epidemics. Previous studies have developed systems capable of detecting aberrations of flu activity, e.g., the onset of flu season, in subtropical cities including Hong Kong and Shenzhen [8,12,32]. Forecasts of other milestones of influenza epidemics (e.g., peak timing) or intensity, however, have not been performed in subtropical and tropical regions, except for the 2009 pandemic [33]. Here we built and tested forecast systems designed to handle the irregular influenza epidemics of the subtropics and tropics. We applied these systems to forecast influenza epidemics in Hong Kong from January 1998 through December 2013, including the 2009 pandemic.

Our findings suggest that to forecast such complex epidemics, the system needs to be sensitive all year round (Fig 1E, multiple epidemics within a year due to different circulating strains/subtypes) yet not generate false alarms for individual strains that are not circulating during some years (e.g., Fig 1A, H1N1 during 2002–2004). Although both the EAKF and PF have proven capable of forecasting influenza epidemics in U.S. cities, additional methods are needed to generate the forecasts for Hong Kong. Specifically, the space reprobing (SR) technique [20] is critical for the SIR-PF system, while adaptive covariance inflation [25,26] and re-initialization are critical for the SIR-EAKF system. Using these new algorithms, we are able to forecast non-seasonal epidemics with accuracy near that of U.S. forecasts, despite the more varied epidemic dynamics of Hong Kong.

In addition to forecast of aggregate incidence time series for all circulating strains, we have also generated forecasts for individual strains, i.e., H1N1 (including both seasonal H1N1 and the 2009 pandemic H1N1), H3N2, and Type B. Forecasts for the individual strains were in general more accurate than those generated for the aggregate epidemics (Fig 4). This finding suggests that strain specific surveillance data indeed provide cleaner signals that enable more accurate forecast. Strain-specific operational real-time forecasts are currently being generated for the U.S. [4].

In our previous work, we focused on forecasting peak timing, i.e., the week with maximum influenza incidence [1,13,19,20]. Here we have expanded the forecast effort to include peak magnitude, onset, and duration. Neither forecast system was able to predict the onset or duration well in advance; however, accuracy predicting outbreak peak magnitude was comparable to that for peak timing. For instance, the SIR-PF system was able to forecast the peak magnitude within 20% of observation with an average accuracy of 37% at 1–3 wk lead and 50% at 0 lead. Further, forecast accuracy increased steadily as ensemble spread decreased: up to 45% for H1N1, 89% for H3N2, and 68% for Type B at 1–3 wk leads (Fig 6, 1^st^ row). This finding suggests that the forecasts provide lead times adequate for the planning of intervention measures. In addition, the forecasts of peak magnitude can be used to inform the scale of response. For instance, the amounts of antivirals and vaccines needed could be assessed based on the predicted peak magnitude.

For this study we opted to use a simple SIR model. This model is a gross simplification of actual transmission dynamics in a population. When used in conjunction with the filter, however, the filtering process, through recursive optimization, partially compensates for model misspecification. As our understanding of influenza transmission dynamics in subtropical and tropical regions improves in the future, more mechanistic and detailed models could be used in conjunction with the filters. For instance, epidemic models that account for the cross-immunity due to prior infections from related strains, age-structured models that account for varying transmission dynamics among age groups, or network models that account for spatial connectivity among sub-regions, could be applied. These more complicated model-filter systems could further improve the forecast performance. Future study will also investigate methods to improve forecast accuracy for onset timing and epidemic duration, both of which are important for public health planning.

In conclusion, we have developed the first prediction systems able to forecast the course of both inter-pandemic and pandemic influenza epidemics in a subtropical locale. These systems can be applied to currently circulating influenza A subtypes and influenza B, as well as aggregate epidemics due to any combination of these influenza strains. The forecast systems are able to predict the peak timing and peak magnitude at least 3 weeks before the predicted peak, with increased accuracy as the ensemble spread decreases.

## Supporting Information

S1 Dataset ILI+ time series for individual strains and combining all influenza strains used in this study. (CSV)

S1 Fig Characteristics of the Hong Kong (HK) influenza epidemics. Each column shows the distributions of peak magnitude (1^st^ row), epidemic duration (2^nd^ row), and time from the onset to the peak (3^rd^ row) for each strain/subtype or all strains combined. The last column also shows the corresponding epidemic characteristic observed in New York City (NYC) for comparison.
(TIF)

S2 Fig Simulation of the Hong Kong ILI+ time series using the SIR-PF *without* space reprobing. Simulations were performed for (A) H1N1, including seasonal and pandemic H1N1, (B) H3N2, (C) influenza B, and (D) all strains. Weekly ILI+ observations are shown as ‘x’; the SIR-PF simulated ILI+ are shown by the blue lines.
(TIF)

S3 Fig Simulation of the Hong Kong ILI+ time series using the SIR-PF *with* space reprobing. Simulations were performed for (A) H1N1, including seasonal and pandemic H1N1, (B) H3N2, (C) influenza B, and (D) all strains. Weekly ILI+ observations are shown as ‘x’. One hundred simulations were run for each time series; mean ILI+ estimates are shown by the blue lines; 95% confidence intervals (CIs) are shown by the grey dashed lines. Note that the 95% CIs are very close to the mean trajectories and are barely visible.
(TIF)

S4 Fig Estimates of population susceptibility using the SIR-PF with space reprobing. Simulations were performed for (A) H1N1, including seasonal and pandemic H1N1, (B) H3N2, (C) influenza B, and (D) all strains. One hundred simulations were run for each time series; mean estimates are shown by the blue lines; 95% confidence intervals are shown by the grey dashed lines.
(TIF)

S5 Fig Estimates of the basic reproductive number using the SIR-PF with space reprobing. Simulations were performed for (A) H1N1, including seasonal and pandemic H1N1, (B) H3N2, (C) influenza B, and (D) all strains. One hundred simulations were run for each time series; mean estimates are shown by the blue lines; 95% confidence intervals are shown by the grey dashed lines.
(TIF)

S6 Fig Estimates of the infectious period using the SIR-PF with space reprobing. Simulations were performed for (A) H1N1, including seasonal and pandemic H1N1, (B) H3N2, (C) influenza B, and (D) all strains. One hundred simulations were run for each time series; mean estimates are shown by the blue lines; 95% confidence intervals are shown by the grey dashed lines.
(TIF)

S1 Movie Weekly 40-week forecasts for H1N1, generated using the SIR-PF forecast system. Each frame shows a two-year time window (month and year shown on the x-axis); ILI+ observations are shown by the ‘x’; the grey vertical line indicates the time of forecast initiation (specified by the week ending shown at the top left corner); fitted ILI+ prior to the forecast are shown by the solid blue line; predicted ILI+ for the following 40 weeks are shown by the dashed blue line.
(MP4)

S2 Movie Same as S1 Movie, but for H3N2. (MP4)

S3 Movie Same as S1 Movie, but for influenza B. (MP4)

S4 Movie Same as S1 Movie, but for aggregate ILI+ epidemic curve caused by one or more of the three influenza strains. (MP4)
