# Supplementary figures and images for: Forecasting Influenza Epidemics in Hong Kong
Source: PLoS Comput Biol. 2015 Jul 30;11(7):e1004383. doi: 10.1371/journal.pcbi.1004383 (PMC4520691; doi:10.1371/journal.pcbi.1004383)

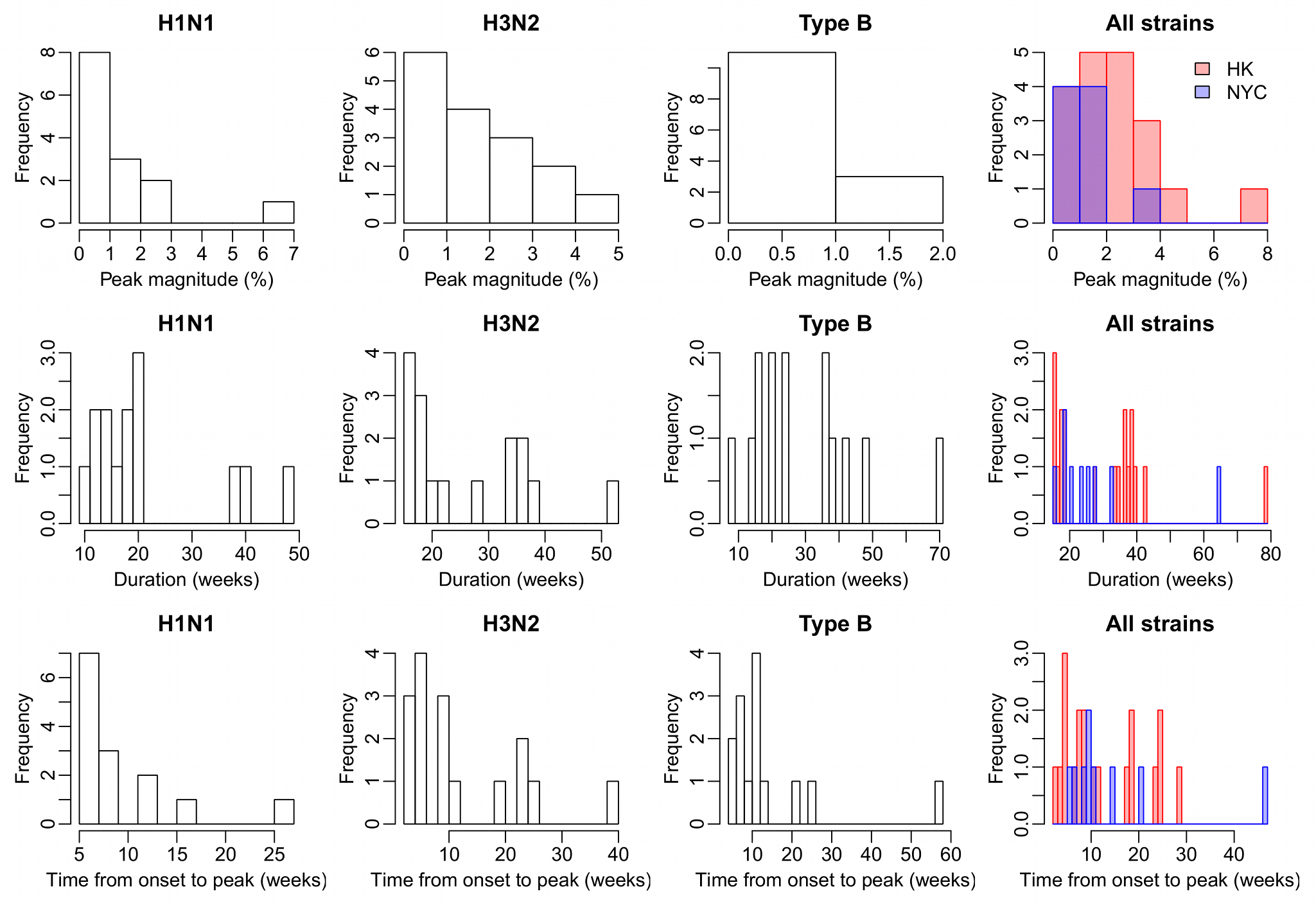

Supplement: S1 Fig — Each column shows the distributions of peak magnitude (1st row), epidemic duration (2nd row), and time from the onset to the peak (3rd row) for each strain/subtype or all strains combined. The last column also shows the corresponding epidemic characteristic observed in New York City (NYC) for comparison. (TIF) [file pcbi.1004383.s002.tif]

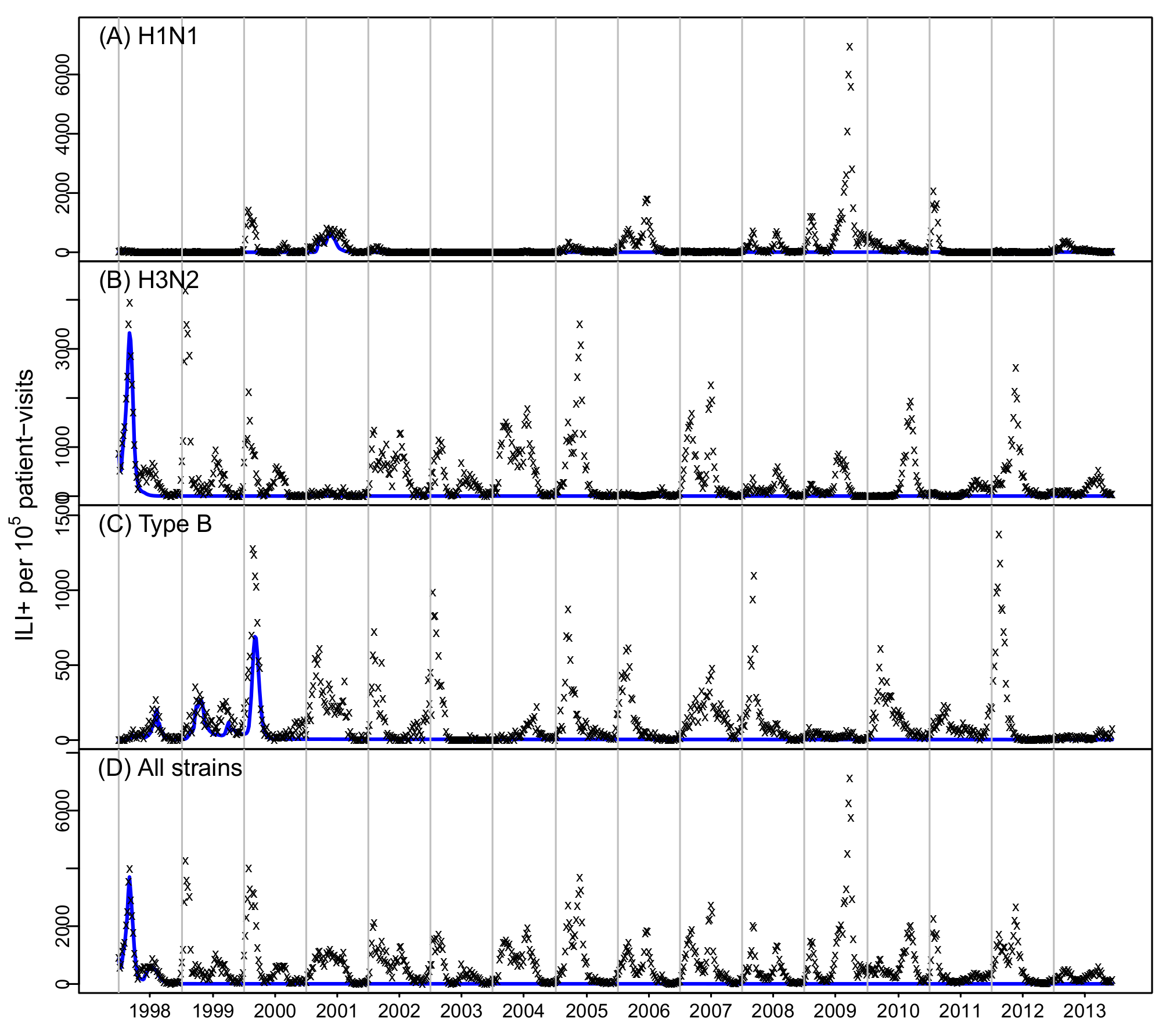

Supplement: S2 Fig — Simulations were performed for (A) H1N1, including seasonal and pandemic H1N1, (B) H3N2, (C) influenza B, and (D) all strains. Weekly ILI+ observations are shown as ‘x’; the SIR-PF simulated ILI+ are shown by the blue lines. (TIF) [file pcbi.1004383.s003.tif]

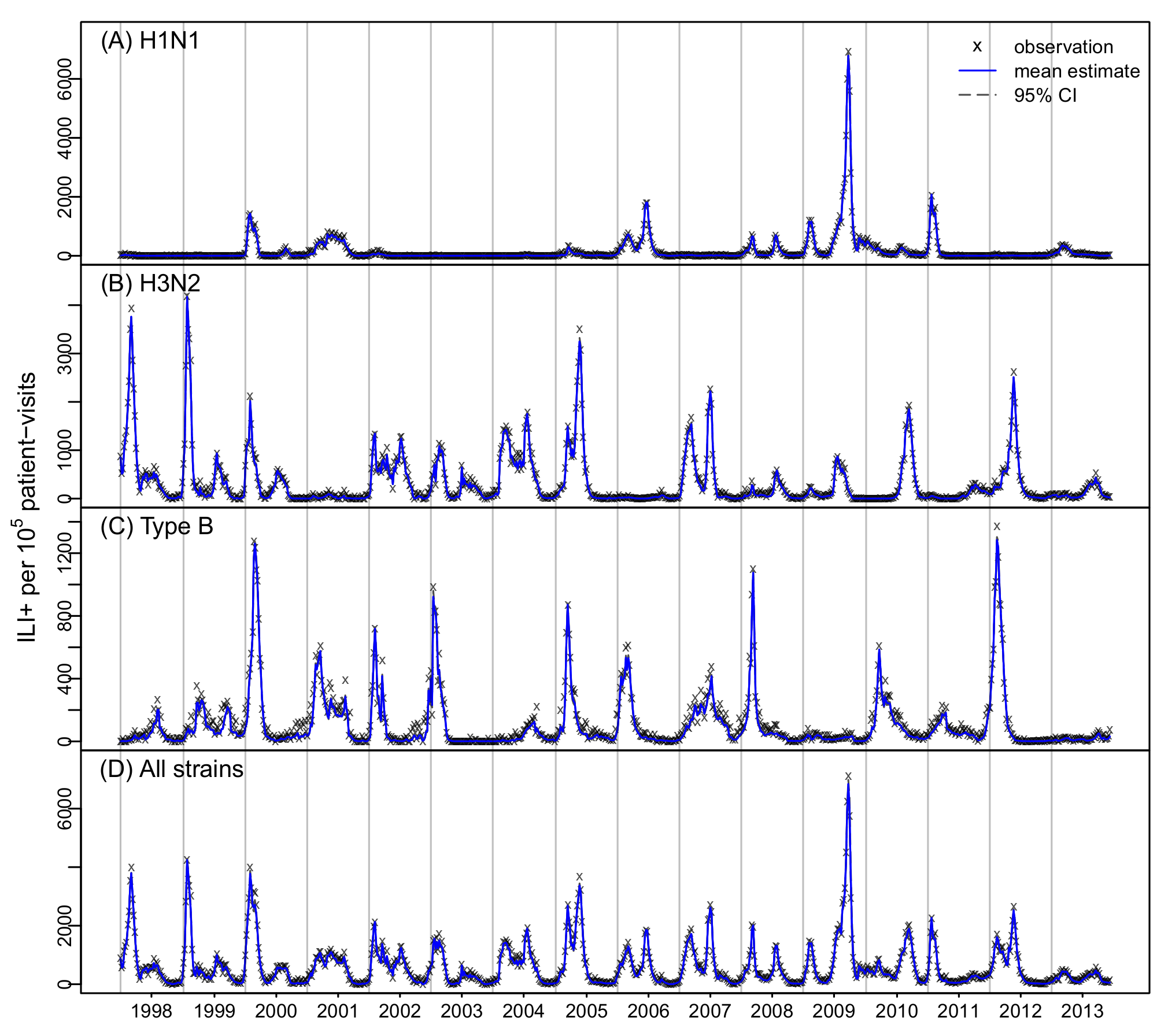

Supplement: S3 Fig — Simulations were performed for (A) H1N1, including seasonal and pandemic H1N1, (B) H3N2, (C) influenza B, and (D) all strains. Weekly ILI+ observations are shown as ‘x’. One hundred simulations were run for each time series; mean ILI+ estimates are shown by the blue lines; 95% confidence intervals (CIs) are shown by the grey dashed lines. Note that the 95% CIs are very close to the mean trajectories and are barely visible. (TIF) [file pcbi.1004383.s004.tif]

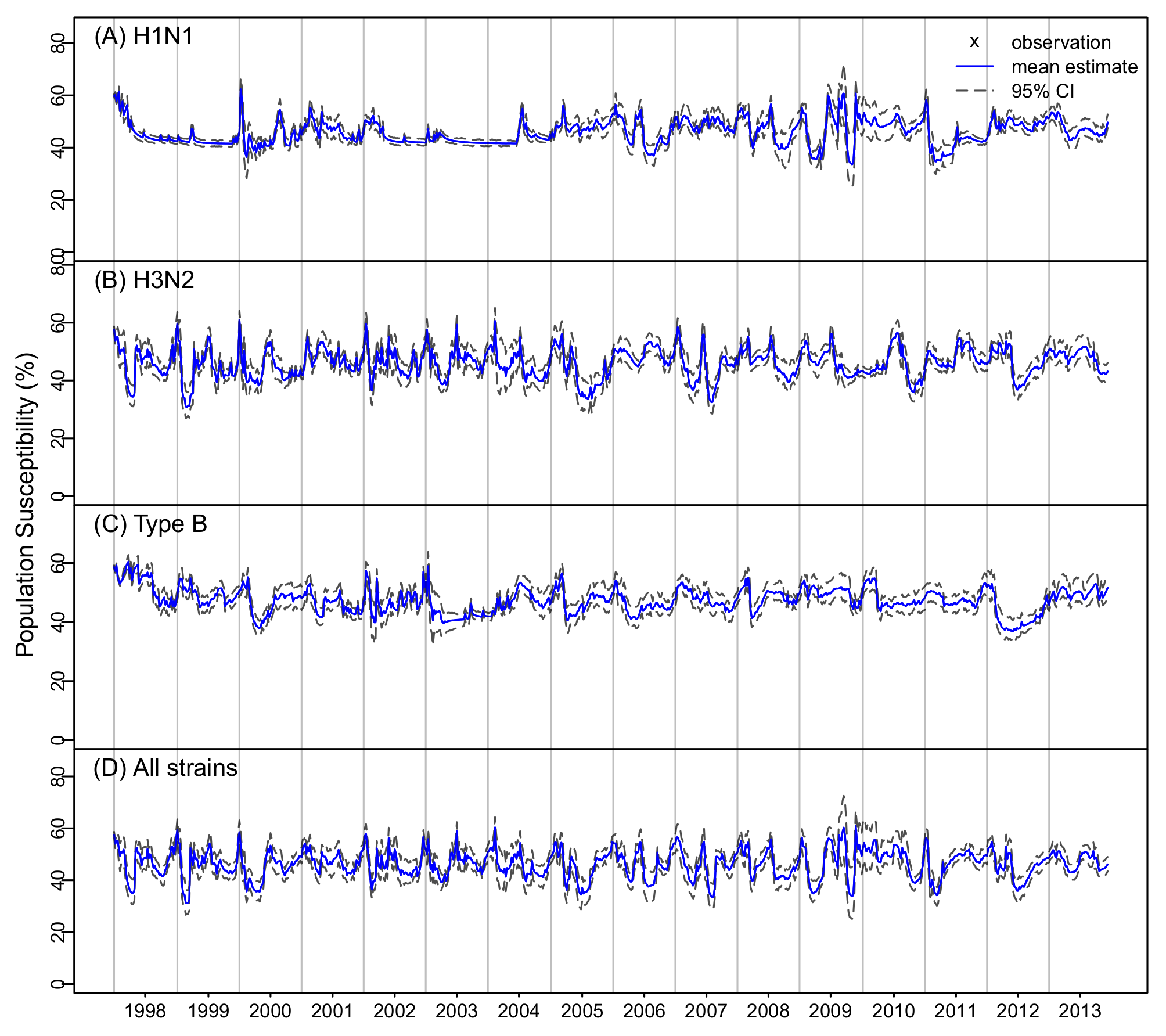

Supplement: S4 Fig — Simulations were performed for (A) H1N1, including seasonal and pandemic H1N1, (B) H3N2, (C) influenza B, and (D) all strains. One hundred simulations were run for each time series; mean estimates are shown by the blue lines; 95% confidence intervals are shown by the grey dashed lines. (TIF) [file pcbi.1004383.s005.tif]

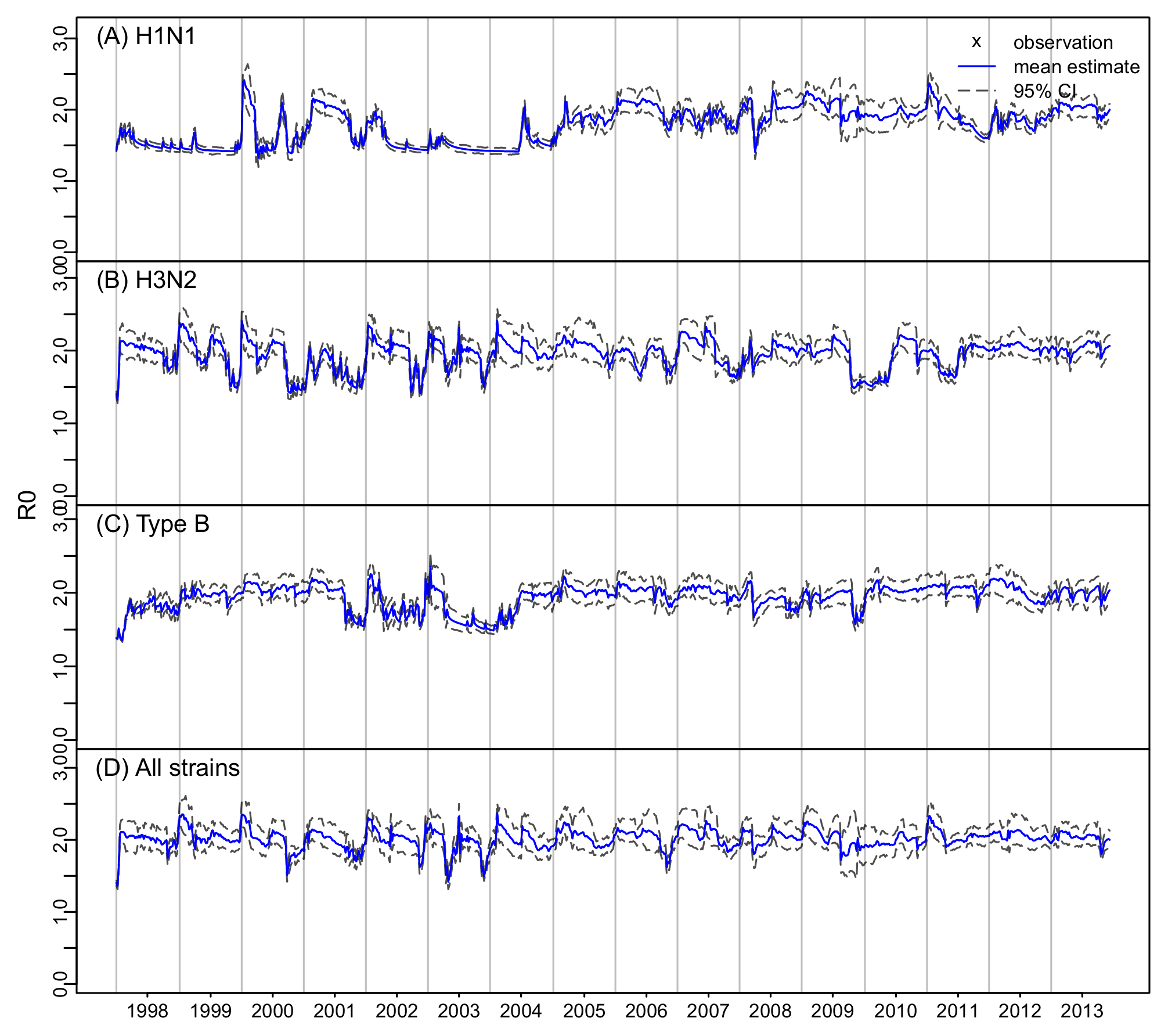

Supplement: S5 Fig — Simulations were performed for (A) H1N1, including seasonal and pandemic H1N1, (B) H3N2, (C) influenza B, and (D) all strains. One hundred simulations were run for each time series; mean estimates are shown by the blue lines; 95% confidence intervals are shown by the grey dashed lines. (TIF) [file pcbi.1004383.s006.tif]

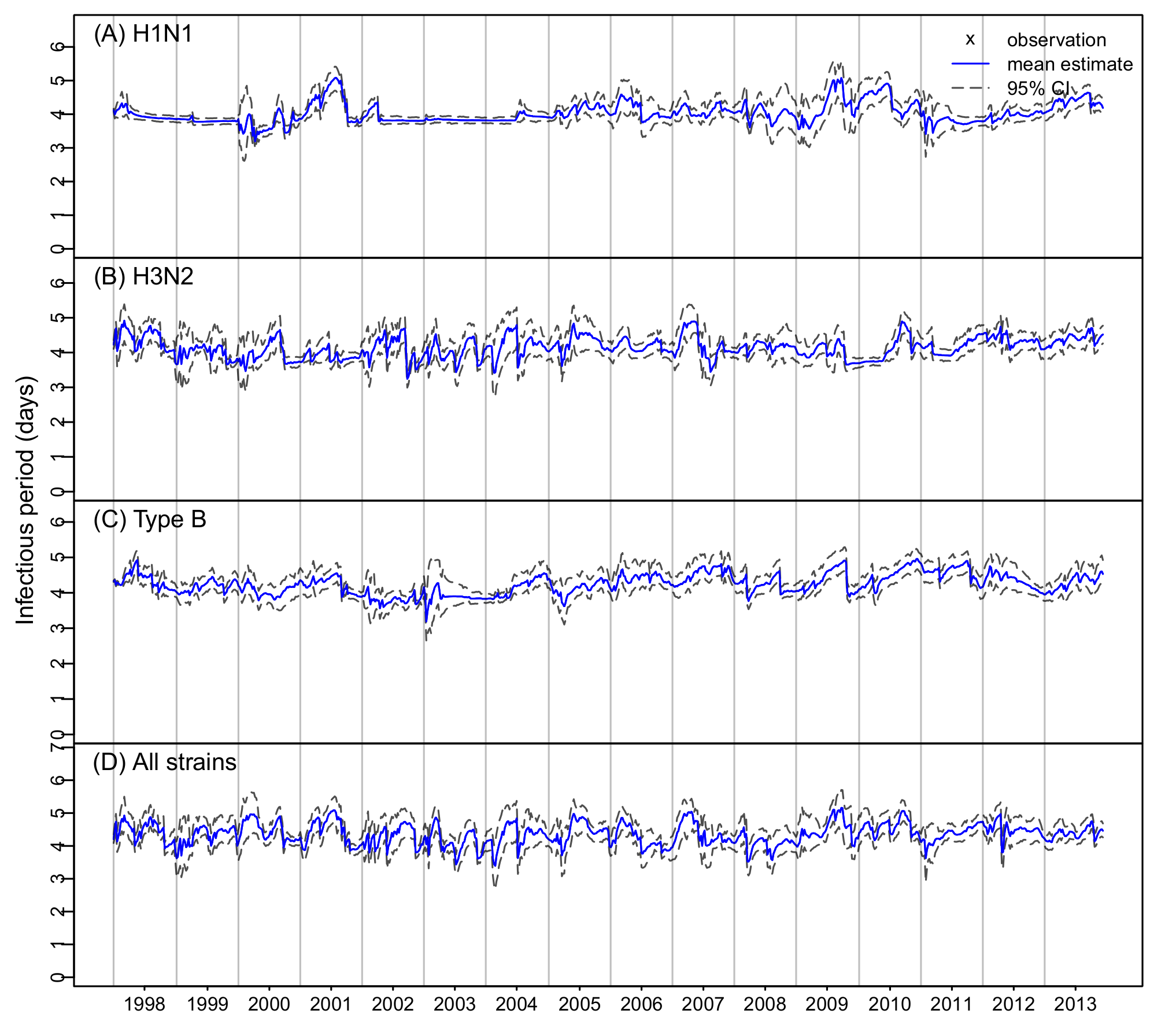

Supplement: S6 Fig — Simulations were performed for (A) H1N1, including seasonal and pandemic H1N1, (B) H3N2, (C) influenza B, and (D) all strains. One hundred simulations were run for each time series; mean estimates are shown by the blue lines; 95% confidence intervals are shown by the grey dashed lines. (TIF) [file pcbi.1004383.s007.tif]
